# Bispecific, T-Cell-Recruiting Antibodies in B-Cell Malignancies

**DOI:** 10.3389/fimmu.2020.00762

**Published:** 2020-05-07

**Authors:** Margaux Lejeune, Murat Cem Köse, Elodie Duray, Hermann Einsele, Yves Beguin, Jo Caers

**Affiliations:** ^1^Laboratory of Hematology, GIGA I3, University of Liège, Liège, Belgium; ^2^Department of Hematology, CHU de Liège, Liège, Belgium; ^3^Department of Internal Medicine II, University of Würzburg, Würzburg, Germany

**Keywords:** bispecific antibodies, leukemia, lymphoma, myeloma, bispecific T-cell engager, BiTE, clinical development, concerns

## Abstract

Bispecific antibodies (BsAbs) are designed to recognize and bind to two different antigens or epitopes. In the last few decades, BsAbs have been developed within the context of cancer therapies and in particular for the treatment of hematologic B-cell malignancies. To date, more than one hundred different BsAb formats exist, including bispecific T-cell engagers (BiTEs), and new constructs are constantly emerging. Advances in protein engineering have enabled the creation of BsAbs with specific mechanisms of action and clinical applications. Moreover, a better understanding of resistance and evasion mechanisms, as well as advances in the protein engineering and in immunology, will help generating a greater variety of BsAbs to treat various cancer types. This review focuses on T-cell-engaging BsAbs and more precisely on the various BsAb formats currently being studied in the context of B-cell malignancies, on ongoing clinical trials and on the clinical concerns to be taken into account in the development of new BsAbs.

## Introduction

The idea of bispecific antibodies (BsAbs) was initially launched in the early 1960s and the first examples were constructed in 1985 (1). Ten years later, a BsAb (anti-CD19 × anti-CD3) was studied in a clinical trial for the treatment of non-Hodgkin’s lymphoma (NHL) (2) and it took until 2009 for the approval of catumaxomab (anti-epCAM × anti-CD3) for the treatment of patients with malignant ascites (3). Advances in protein engineering enable the creation of BsAbs with specific mechanisms of action and clinical applications (4). Although catumaxomab was withdrawn from the market in 2017 for commercial reasons, the excellent clinical results of the bispecific T-cell engager (BiTE), blinatumomab (anti-CD19 × anti-CD3) (5), have renewed the interest and investment in BsAb development.

## Bispecific Antibodies

Bispecific antibodies are designed to bind to two different antigens (Ag) or epitopes. These Ags can be present on the same cell, thereby improving the selectivity and binding kinetics of these antibody (Ab) formats. Most BsAbs are developed to bind different targets on different cells, which expand their potential applications. In immunotherapy, they are used to improve tumor cell eradication by bringing cytotoxic cells [T-cells or natural killer (NK)-cells] directly in contact with tumor cells. Given their potential economic value, the pharma industry has taken over their biotechnical development resulting in more than 100 different formats that have been designed (6). This review tries to focus on different T-cell recruiting formats that have been developed in the treatment of B-cell malignancies.

**FIGURE 1:**
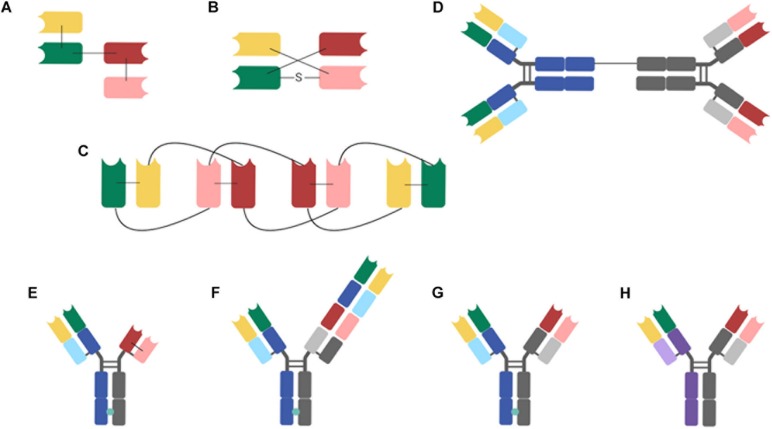
BsAb formats studied for hematological B-cell malignancies **(A)**, BiTE (Tandem scFvs); **(B)** DART; **(C)** TandAb (Tandem diabodies); **(D)** BAT; **(E)** TDB: Xmab (scFv-Fab IgG); **(F)** TCB: CrossMAb; **(G)** TDB: DuoBody; **(H)** TriFAb (Rat-mouse hybrid IgG). The different antibody domains are as follows: green, variable region of heavy chain 1 (VH 1); red, variable region of heavy chain 2 (VH 2); yellow, variable region of light chain 1 (VL 1); pink, variable region of light chain 2 (VL 2); light purple, constant region of light rat chain; dark purple, heavy chain of immunoglobulin G2b (IgG2b); light blue and light gray, constant regions of light mouse chain; dark blue and dark gray, heavy chains of mouse IgG2b; turquoise circles, Knob-in-Hole (KiH) BiTE, bispecific T-cell engager; DART, dual-affinity re-targeting; Fab, Fab region; S, disulfure; scFv, single-chain variable fragment; TandAb, tandem diabody; TDB, T-cell-dependent bispecific antibody; TriFAb, trifunctional antibody, triomab.

Effector cell-engaging BsAbs are generally made up of an effector cell-binding domain linked to a tumor Ag-binding fragment. The final format can be made of various known Ab fragments such as single-chain variable fragment (scFv), heavy chain variable domain (VH), light chain variable domain (VL), variable region of a heavy chain of a heavy chain only Ab (VHH), diabody, etc.; or resemble the general architecture of immunoglobulins (Ig). Such fragments provide advantages and disadvantages according to their specific characteristics and properties. Therefore, selection of Ab fragments require careful evaluation, in order to create the most suitable BsAbs for the desired applications (4, 7). One single format is probably not suitable for all applications and BsAbs are generated according to desired characteristics. They differ in terms of size, valency, flexibility, distribution of their pharmacological properties, etc. The two most common forms of BsAbs are the IgG-based and Ab-fragment based formats. IgG-Based BsAb contain an Fc region that helps the stability of the BsAb and the production and purification procedures. Some of the formats of BsAbs currently used for hematological cancers are described in Tables 1, 2 and these various formats are shown in Figure 1.

**TABLE 1:** Ab formats used for hematological cancers: Bispecific antibodies IgG-like.

Name/Platform	Firm	Characteristics	Heavy chain engineering		Light chain engineering	Fc domain	Production	Remarks	References
			
			“Knob-in-hole” technology	Other strategies					
BsAb armed activated T-cells (BAT)	Mostely academic	Combination of an mAb targeting the tumor Ag with an mAb targeting the effector cells	No	No	No	Functional Fc	Chemical heteroconjugation of 2 mAbs	Combined with *ex vivo* activated T-cells	161
CrossMab	Roche	Exchange of either the constant domain, variable domains or the whole Fab fragment	Yes	Electrostatic steering	Crossover of an existing fragment without the need for the identification of common light chains	Fc part without effector function	Almost natural, full-sized humanized IgG1 antibody	Not immunogenic, also applied to 2 + 1 and 2 + 2 formats	162, 163
Veloci-Bi	Regeneron	Common light chain approach combined with mutation of protein A binding site for improved purification	No	Selection of correct heterodimers by Protein A affinity chromatography using a new protein A resin	Use of heavy chains that employ identical light chain	Fc part without effector function	Recombinant production, purification enables identification of correct heterodimers	Not immunogenic	164
SEEDbodies		Specific pairing through the design of alternating segments from human IgA and IgG	No	Strand-exchange engineered domain: interdigitating β-strand segments of human IgG and IgA C_H_3 domains	Additional engineering for correct heavy-to-light chain pairing	Fc part without effector function	Recombinant production	SEEDbodies assure correct Heavy chain pairing, but additional engineering of light chains can be necessary	165
Biclonics	Merus	Charge pairs in the CH3 that favor heterodimerization	No	Introduction of charged residues at different positions within the Fc part	Fab fragment consisting of common light chain fragments	Fc part without effector function	VH genes cloned in the backbone IgG1; Recombinant production of full IgG	/	166, 167
XmAb	Xencor	Typically, scFv fused to one Fc instead of Fab fragment to enable bispecificity	Yes	Set of minor and precise changes to the Fc region leading enhanced heterodimerization Improved purification procedure	Different formats exist: Fab or ScFV	Fc part without effector function	Recombinant production and purification by l protein A affinity chromatography	Full-sized humanized IgG1 Ab, nearly identical to natural Ab (similar structure and sequence)	168
Duobody	Genmab	Controlled Fab-arm exchange (cFAE) from two parent homodimeric antibodies	Yes	Fc silent mutations	Separate expression and purification of the 2 component antibodies followed by assembly into BsIgG	Fc activity can be retained or silenced depending on the characteristics desired	Almost natural, full-sized humanized IgG1 antibody	Full-sized humanized IgG1 Ab, minimal modifications to the native Ab structure	169
TriFAb (Trifunctional Ab)	TRION	Produced from two half antibodies from parental mouse IgG2a and rat IgG2b isotypes	No	/	Species−restricted heavy/light chain pairing	Fc part with effector function	Produced using the quadroma technology and captured by protein A affinity chromatography	Trifunctional ≥ Highly immunogenic and toxic (CRS)	170

**TABLE 2:** Ab Formats used for hematological cancers: Bispecific antibodies with single chain formats.

	Characteristics	Molecular Weigth	Half life	Linker	Administration	Remarks	References
BiTE	2 scFv fragments, connected by flexible linker peptides	∼55 kDa	2 h	15–amino acid (G4S1)3 (single-letter amino acid code) linker	Continuous infusion	Rely exclusively on effector-tumor synapse formation	171
BiKE	BiKEs: 2 scFv fragments, connected by flexible linker peptides are similar in design to BiTEs but they target CD16 on NK-cells	58–60 kDa	ND	20-amino acid segment of human muscle aldolase	ND	Not immunogenic, further expansion of NK-cells (TriKE)	172, 173
TriKE	TriKEs consist of a BiKE into which IL-15 was subsequently sandwiched	∼96 kDa	ND	Human IL-15 with N72D substitution, flanked by two flanking sequence	ND	Mutated form of IL-15 expands NK-cells	173
Diabodies	A single−chain format based on 2 peptides, each one contains a heavy chain variable region (VH) for an Ag recognition site paired with a light chain variable region (VL) of a second Ag recognition site	58 KDa	2 h	15 amino acids with sequence GGGGSGGRASGGGGS	Frequent injections or infusions	Variants of diabodies consist of dual-affinity retargeting molecules (DART) or tetravalent constructs that combine two diabodies (TandAb)	174

### Bispecific Antibodies IgG-Like

The Fc domain of an Ig facilitates BsAb purification, improves solubility and stability, extends their *in vivo* half-life (8) and activates several immune cells. When its effector functions are maintained, this Fc region will induce Ab-dependent cell-mediated cytotoxicity (ADCC) by recruiting NK-cells and/or macrophages and complement-dependent cytotoxicity (CDC) by binding the complement (4, 8).

Preferably, CD3-targeting BsAbs require the complete suppression of the Fc-mediated effector functions in order to maximize therapeutic efficacy and to minimize off-target toxicity because binding of Fc to Fc gamma receptor (FcγR) leads to activation of immune effector cells. In reality, the majority of the CD3-targeting BsAbs, currently in clinical practice, have Fc domains with reduced binding activity to FcγR or are BsAb fragments intentionally without the Fc region (9).

However, IgG-like BsAbs composed of two different heavy chains and two different light chains are difficult to produce. The heavy chains of the Bsab can form homodimers (described as heavy chain-pairing problem) and also the light chains can pair to the incorrect heavy chains (light chain-pairing problem). Different solutions have been proposed to avoid these undesired mispairs and some of them are integrated in Table 1. A major progress in this field was the development of the “knobs-into-holes” (KiH) strategy that consisted of introducing large amino acid side chains into the CH3 domain of one heavy chain that fit into an appropriately designed cavity in the CH3 domain of the other heavy chain (10).

### Bispecific Antibodies Without Fc Region

BsAbs lacking an Fc region can be produced by linking two different single-chain antibodies with a linker. Their Ag-binding part contain only the variable regions of the heavy and light chains connected to each other by a linker (Table 2). They are smaller than the bispecific molecules with an Fc region, and this reduced size results with increased tissue penetration, but also fast renal elimination resulting in a short plasma half-life. This reduced circulation time requires more frequent administrations or continued infusion (11, 12). The half-life can be extended using different engineering technologies, such as coupling to inert polymers (polyethylene glycol) (13) adding an Fc part (14), attaching an albumin-binding part (15) or even immunoglobulin-binding domains (16). Companies are currently introducing these half-life extended BsAb in order to limit the administration frequency and improve patients’ comfort. Prospective clinical studies will investigate the efficacy and toxicity of these conjugated BsAbs and allow a comparison with the original BsAbs (e.g., AM701, an anti CD3 × BCMA BiTE) is a half-life extended version of AMG420 that showed promising results in the first phase I trial).

## Recruitment of Effector Cells

### Main Ag for Targeting T-Cells: CD3

BsAb constructs guide immune effector cells to tumor cells by cell-specific receptors such as CD3 on T-cells or CD16 on NK-cells. Currently, approximately half of the evaluated BsAbs by clinical trials are BsAbs that recruit T-cells (17). Their mechanism of action is based on the activation of T-cells by binding CD3ε in the T-cell receptor (TCR) complex irrespective of major histocompatibility complex (MHC) restriction or TCR epitope specificity. Although required for their anti-cancer activity, the binding to the antigen may lead to an excessive immune reaction with activation of bystander immune cells and non-immune cells that finally results in a cytokine release syndrome (CRS).

Most T-cell engaging BsAbs aim to bind CD3ε to guide T-cells to the target cells. An alternative Ag, CD5, has been previously explored (18) but the observed responses were inferior to those obtained with CD3ε-binding BsAbs. Unfortunately, CD3 will recruit different types of T-lymphocytes (including immune-suppressive ones) that can limit their efficacy. For example, Duell et al. (19) showed that blinatumomab also activates regulatory T-cells (Tregs), who inhibit cytotoxic T-cell proliferation, thereby preventing tumor cell destruction. As a result, usage of NK-cells instead of T-cells draw attention in clinical development (see Table 3) (17).

**TABLE 3:** Clinical development of BsAbs (selected trials).

Names (Sponsors)	Targets (diseases indications)	Format	Phase (NCT#)	References
**T-cell redirection**				
AMG420, BI 836909 (Boehringer Ingelheim, Amgen)	CD3 × BCMA (MM)	Tandem scFv (BiTE)	Phase I (NCT02514239, NCT03836053)	102, 175
AMG701 (Amgen)	CD3 × BCMA (MM)	Tandem scFv-scFc(G1) (HLE-BiTE) Possibly Fc-silencing	Phase I (NCT03287908)	176
CC-93269, EM901 (Celgene)	CD3 × BCMA (MM)	Fab-Fc(G1) × Fab-Fab-Fc(G1) (CrossMab in the 2 + 1 format) Possibly Fc-silencing	Phase I (NCT03486067)	103
JNJ-64007957 (Janssen)	CD3 × BCMA (MM)	Hetero H, HL exchanged IgG4 (DuoBody) Possibly Fc-silencing	Phase I (NCT03145181)	177
PF-06863135 (Pfizer)	CD3 × BCMA (MM)	Hetero H, HL assembly IgG (DuoBody) Possibly Fc-silencing	Phase I (NCT03269136)	106
REGN5458 (Regeneron)	CD3 × BCMA (MM)	Hetero H, cL IgG4 Possibly Fc-silencing	Phase I/II (NCT03761108)	178
AMG424, Xmab13551 (Amgen)	CD3 × CD38 (MM)	Fab-Fc(G1) × scFv-Fc(G1) (Xmab) Possibly Fc-silencing	Phase I (NCT03445663)	97
GBR 1342 (Glenmark)	CD3 × CD38 (MM)	Fab-Fc(G1) × scFv-Fc(G1) (Xmab) Possibly Fc-silencing	Phase I (NCT03309111)	98, 179
RG6160, RO7187797, BFCR4350A (Genentech)	CD3 × FcRH5 (CD307) (MM)	Hetero H, HL assembly IgG1, IgG assembled from half-antibodies	Phase I (NCT03275103)	35
JNJ-64407564 (Janssen)	CD3 × GPRC5D (MM)	Hetero H, HL exchange IgG4 (DuoBody) Possibly Fc-silencing	Phase I (NCT03399799)	109
Vibecotamab, Xmab14045 (Xencor)	CD3 × CD123 (B-ALL, AML, CML)	Fab-Fc(G1) × scFv-Fc(G1) (Xmab) Possibly Fc-silencing	Phase I (NCT02730312)	180, 181
A-319 (Generon)	CD3 × CD19 (B-cell lymphoma)	scFv-Fab (ITab)	Phase I (NCT04056975)	182
MGD011, JNJ-64052781 (Janssen)	CD3 × CD19 (NHL, B-ALL, CLL)	DART	Phase I: Withdrawn (NCT02743546)	85
AFM11 (Affimed)	CD3 × CD19 (ALL, NHL)	Tandem diabodies (TandAb)	Phase I: Suspended (NCT02106091 and NCT02848911)	86
AMG562 (Amgen)	CD3 × CD19 (NHL)	Tandem scFv-scFc(G1) (HLE-BiTE) Possibly Fc-silencing	Phase I (NCT03571828)	183
Blinatumomab, Blincyto, MT103, MEDI-538, AMG103 (Amgen)	CD3 × CD19 (B-ALL, NHL, MM)	Tandem scFv (BiTE)	Marketed (ALL), Phase I/II [NCT01741792 et NCT02811679 (NHL), NCT03173430 (MM)]	5, 83, 184, 185
GEN3013 (Genmab)	CD3 × CD20 (NHL)	Hetero H, HL exchanged IgG1 (DuoBody) Possibly Fc-silencing	Phase I/II (NCT03625037)	186
Mosunetuzumab, RG7828, RO7030816, BTCT4465A (Genentech)	CD3 × CD20 (CLL, NHL)	Hetero H, HL assembly IgG1, IgG assembled from half-antibodies Possibly Fc-silencing	Phase I/II (NCT03677141 and NCT03677154)	187, 188
Plamotamab, XmAb13676 (Xencor)	CD3 × CD20 (CLL, NHL)	Fab-Fc(G1) × scFv-Fc(G1) (Xmab) Possibly Fc-silencing	Phase I (NCT02924402)	189
REGN1979 (Regeneron)	CD3 × CD20 (ALL, CLL, and NHL)	Hetero H, cL IgG4 Possibly Fc-silencing	Phase I/II (NCT03888105, NCT02290951)	90, 91
RO7082859, RG6026, CD20-TCB (Hoffmann-La Roche)	CD3 × CD20 (NHL)	Fab-Fc(G1) × Fab-Fab-Fc(G1) (CrossMab in the 2 + 1 format) Possibly Fc-silencing	Phase I (NCT03075696)	93
FBTA05, Lymphomun (Technical University of Munich)	CD3 × CD20 (CLL, NHL)	Trifunctional Ab (TriFAb)	Phase I/II (NCT01138579): Terminated	87, 89, 170
CD20Bi (Barbara Ann Karmanos Cancer Institute)	CD3 × CD20 (NHL)	BAT	Phase I (NCT00244946)	190, 191
**NK-cell redirection**				
AFM13 (Affimed)	CD16A × CD30 (NHL, HL)	Tandem diabodies (TandAb)	Phase I/II (NCT02321592, NCT03192202 and NCT04101331	24, 192
**Immune cell redirection**				
INBRX-105 (Inhibrx)	PD-L1 × 4-1BB (NHL, HL)	Tandem VHH-Fc(G1) Possibly Fc-silencing	Phase I (NCT03809624)	193
**Targeting tumor heterogeneity**				
OXS-1550, DT2219ARL (Masonic Cancer Center, University of Minnesota)	CD19 × CD22 (B-cell lymphoma and leukemia)	Tandem scFv fusion protein (BiTE fused to modified diphtheria toxin)	Phase I/II (NCT02370160, NCT00889408)	132, 194
**Targeting multiple checkpoints**				
MGD013 (Macrogenics)	PD-1 × LAG3 (Solid and Hematological malignancies)	Tandem domain-exchanged Fv-Fc(G4) (DART-Fc)	Phase I (NCT03219268)	142
KN046 (Alphamab)	PD-L1 × CTLA4 (Solid and hematological malignancies)	Hetero H, cL IgG1	Phase I (NCT03733951)	195
**Targeting checkpoint and tumor antigen**				
TG-1801, NI-1701 (TG Therapeutics)	CD47 × CD19 (B-cell lymphoma)	cH IgG1 (κλ body)	Phase I (NCT03804996)	196

*Data available as of November 05, 2019. Molecules are classified based on target Ags. ALL, acute lymphoblastic leukemia; AML, acute myeloid leukemia; B-ALL, B-cell acute lymphoblastic leukemia; BAT, Bispecific antibody armed activated T-cells; BCMA, B-cell maturation antigen; BiTE, bispecific T-cell engager; CLL, chronic lymphocytic leukemia; CML, chronic myeloid leukemia; CTLA4, cytotoxic T-lymphocyte-associated protein 4; DART, dual-affinity re-targeting; Fab, antigen-binding fragment; FcRH5, Fc receptor homolog 5 (CD307); GPRC5D, G protein-coupled receptor family C group 5 member D; H, heavy; HL, Hodgkin lymphoma; HLE, half-life extended; Ig, immunoglobulin; ITab, immunotherapy antibody; L, light; LAG3, lymphocyte-activation gene 3; MM, multiple myeloma; NHL, non-Hodgkin lymphoma; NK, natural killer; PD-1, programmed cell death 1; PD-L1, programmed cell death 1 ligand; scFc, single-chain Fc fragment; scFv, single-chain variable fragment; TandAb, tandem diabody; TriFAb, trifunctional antibody, triomab; VHH, heavy chain-only variable domain.*

CD3-based BsAbs targeting T-cells also demonstrated other disadvantages, such as (1) potentially high toxicity, particularly for targets with wide tissue expression; (2) partial tumor destruction and the development of resistance to treatment due Ag escape (8) and rapid and powerful activation of a large pool of T lymphocytes that is no longer counterbalanced by TCR regulation (20, 21). The interest in this type of BsAbs renewed after the first clinical results obtained with blinatumomab (see section “BiTE anti-CD19 – CD3”) (22). Impressive responses were observed at very low doses in patients with NHL who received blinatumomab via a continuous intravenous infusion to reach the desired minimum concentrations (22). In addition, an exceptional complete response rate of 43% was reported in the first studies on relapsed/refractory (r/r) acute lymphoblastic leukemia (ALL) (23).

### Main Ag for Targeting NK-Cells: CD16A

An alternative to T-cell usage consists in activating and directing NK-cells to malignant cells. Compared to T-cells, NK-cells are not subjected to HLA restriction. In addition, NK-cell therapies may be better tolerated by patients than their T-cell counterparts (24). Several receptors capable of activating the cytotoxic function of NK-cells have already been described, notably CD16, NKp30, NKp46, NKG2D and DNAX Accessory Molecule-1 (DNAM-1) (25, 26). Contrary to other activating receptors present in human NK-cells, CD16 can strongly trigger activation without co-stimulatory receptors. There are two isoforms of CD16 in humans, CD16A and CD16B, having a low affinity receptor for IgG Fc domain. CD16A is expressed in NK-cells, macrophages and placental trophoblasts, whereas CD16B is expressed in neutrophils. Only the CD16A isoform is capable of triggering both IL-2 secretion and tumor cell destruction (27).

Despite its advantages, CD16 is often cleaved on the surface of NK-cells by a disintegrin and metalloptroteinase-17 (ADAM17) which likely results in a decrease in the activities mediated by this receptor (28). To address this concern, combining a BsAb and ADAM17 inhibitor was evaluated and showed improved therapeutic efficacy (29). An alternative solution is targeting other receptors on the NK–cells, alone or in parallel to CD16. Recently, the group of E Vivier showed the increased cytotoxic effect of targeting two activating receptors, NKp46 and CD16, on NK-cells (30).

Lastly, in addition to directing the cytotoxicity of the NK-cells, improvements were made to their survival and proliferation. IL-15 was incorporated into a Bispecific Killer cell Engager (BiKE) structure to create a Trispecific Killer cell Engager (TriKE) which was confirmed to have the capability to enhance NK-cell cytotoxicity with improved survival and proliferation *in vitro* (31).

## Binding to Tumor Cells

Various parameters will influence the effectiveness of the BsAbs activity. The major factors that determine whether an Ag is a good target include (1) tumor-specificity and absence on healthy tissues (32), (2) prevalence and level of expression on tumor cells (32), (3) potential expression on malignant precursor or stem cells (33), and (4) low levels of circulating, soluble forms. Moreover, the cytotoxic potential of BsAbs is affected by the target Ag size and the distance between the epitope and the target cell membrane (34, 35). For example, if the distance between the epitopes is large, inhibitory molecules can interfere with the formation of the synapse (35). To achieve optimal effector cell activation, the affinity of the monoclonal Ab, the location of the target epitope in the antigen (Ag) and the Ag density on the surface of the target cells must be taken into account (10).

Furthermore, the low number of truly tumor-specific cell surface molecules limits the use of BsAbs in to cancers where the target Ag is highly overexpressed in malignant cells compared to healthy cells and when the related toxicity toward healthy cells is clinically tolerable (36). Most BsAbs in clinical development target well-known B-cell Ags, particularly the CD19, CD20, CD38, CD123, or B-Cell Maturation Ag (BCMA). These targets are generally also expressed by normal plasma cells and B-cells. Nevertheless, depletion of these cells can be tolerated without inducing serious clinical side effects (17). In addition, these targets are specific for hematopoietic lineage and are not expressed in other normal tissues, which helps to reduce off-tumor activity and side effects.

## B-Cell Malignancies

**FIGURE 2:**
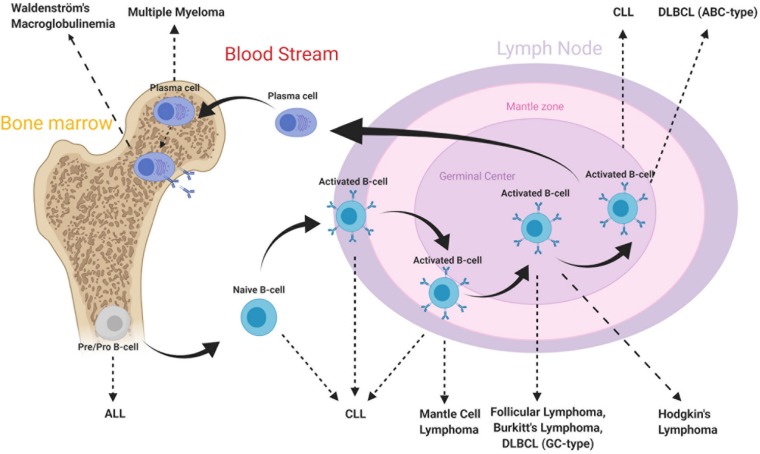
B-cell subtypes and associated malignancies. From Hematopoietic stem cell differentiation into myeloid and lymphoid lineages. After Ag-binding, B-lymphocytes further mature in lymphoid tissues where they undergo various morphological, genetic, and chromosomal alterations. As a consequence, various cell surface Ags reside on cell membrane along maturation process. Disruptions in these mechanisms may lead to the development of malignancies. The B-cell malignancies are divided into subgroups based on location, subtype and activation state of B-cells. This figure is adapted from (70, 159).

**FIGURE 3:**
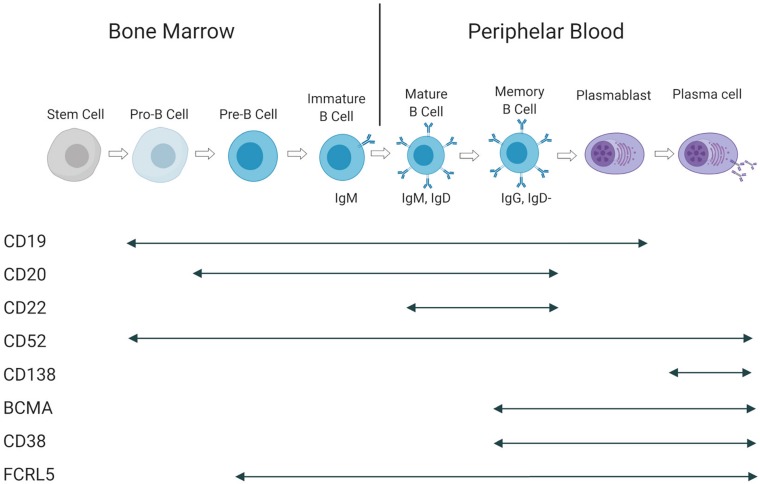
Antigen expression during B-cell maturation. Cell surface Ags and their presence at in different B-cell subtypes. This figure is adapted from (160).

The B-cell subtypes and the various associated malignancies as well as the different Ags expressed in the B-cell lineage are shown in Figures 2, 3.

### Acute Lymphoblastic Leukemia

Acute lymphoblastic leukemia (ALL) is a hematological malignancy induced by proliferation and accumulation of immature lymphoblasts in various tissues. It is seen in both pediatric and adult patients, showing a bimodal distribution (37). While young patients have a good prognosis, the outcome for adults can be dismal (38, 39). Its prognosis depends further of other factors, such as age, chromosomal abnormalities, genetic alterations and the implicated cell lineage. Although, ALL can be derived from NK-cell, T-cell and B-cell lineages, the majority of the disease is associated with B-cell precursors (40, 41). Chromosomal abnormalities play a critical role in development of ALL. The Philadelphia chromosome (Ph) or translocation *t*(9;22), is a critical anomaly that determine the characteristic of the disease, yielding poor prognosis (42, 43). Initially, patients are diagnosed based on the abundance of lymphoblasts (>20%) in bone marrow or blood (44). Since ALL is associated with premature B-cells, B-cell specific differentiation markers; CD19, CD20, and CD22, are highly associated Ags that are used for diagnosing and targeting with immunotherapeutic agents (40, 45).

### Chronic Lymphocytic Leukemia

Chronic lymphocytic leukemia (CLL) is a hematologic disorder defined by accumulation of monomorphic mature B-cells within blood, bone marrow, lymph nodes, and spleen (46). It is observed with a median age at diagnosis of 70 and male:female ratio of 1.5 (47).

Chronic lymphocytic leukemia progression is driven by various genetic abnormalities. Somatic mutations, such as TP53, BIRC3, NOTCH1, ATM, and SF3B1 disrupt pathways including DNA damage, cell cycle control, NOTCH signaling and mRNA processing (48–50). Deletion of chromosome 13 (loss of miR-15a and miR-16-1) and trisomy 12 are the most common chromosomal aberrations observed in CLL, triggering tumorigenesis. Secondary abnormalities are observed at the later stages of the disease, causing resistance to therapy. Essentially, the presence of mutations or deletions in the p53 gene and the mutation status of the immunoglobulin heavy-chain variable region gene (IGH) are strong indicators of poor prognosis (51–54).

Chronic lymphocytic leukemia is well characterized by the expression of CD5 and CD23 along with B-cell markers CD19, CD20, together with high abundance of a single light chain (κ or λ), due to clonal B-cell amplification (46). The diagnosis is obtained by immunophenotyping and blood count of B-cells. If monoclonal B-cells are more than 5000 cells per μL, the diagnosis of CLL is retained (55).

### Multiple Myeloma

Multiple myeloma (MM) is an incurable malignancy, caused by monoclonal proliferation of non-functional plasma cells in the bone marrow (56). The median age at diagnosis is 69 years with median overall survival of 8.5 years for transplant-eligible patients (57). Although good response rates are observed with initial therapy, the disease relapses and no longer responds to therapy, causing poor prognosis (56).

Multiple myeloma is characterized by the secretion of monoclonal immunoglobulins or light chains (described as M-protein). Initially, it is a benign disorder where 5 to 10% can evolve into a symptomatic malignancy (58, 59). This progression is driven by a clonal evolution within malignant plasma cells. The genomic infrastructure of MM is highly heterogeneous. Although, the events leading to MM transformation are unclear, numerous genetic abnormalities contribute to disease progression. Disruption of regulation of cyclin D and IgH proteins, including translocations *t*(11;14) and *t*(4;14), are common chromosomal abnormalities observed in early stages, together with hyperdiploidy located in odd chromosomes (60, 61). During progression, as the disease advances, the genetic stability decreases. Additional aberrations, such as chromosomal loss/gains, somatic mutations (KRAS, NRAS, and TP53), hypermethylation and more translocations (MYC), trigger further oncogenic events (62, 63).

An initial diagnosis is assessed by monoclonal protein level, bone marrow biopsy, radiologic imaging and is based on the presence of symtoms (annotated by the acronym CRAB: hypercalcemia, renal failure, anemia and bone lesion) (64, 65). Furthermore, the disease stage can be obtained by International Staging System (ISS) (66), revised on 2015 with additional genetic risk factors (67). Although there is no specific gene marker for MM, Ags such as CD38, BCMA, and CS1/SLAMF7, are currently targeted by immunotherapeutic strategies (68).

### Non-Hodgkin Lymphomas

Non-Hodgkin lymphomas are B-cell malignancies that are primarily located in lymph nodes. The disease progression is driven by precursor lymphocytes, where 85% of the cases emerge from B-cell precursors (69). The 5-year survival rates vary highly, from 30% to 86%, among the subtypes of NHL (70). These subtypes are mainly categorized into two groups. Aggressive lymphomas are rapidly evolving entities with a high tumor cell proliferation rates, but potentially curable when responding to high-dose chemotherapy. In contrast, indolent subtypes represent low grade lymphomas and are incurable (71).

Specific translocations enhance the expression of oncogenic proteins and disrupt DNA damage control mechanisms and will finally result in the development of various NHL subtypes (69). To target these cells, cell surface Ags CD19, CD20 and CD30 are widely used targets (72). The diagnosis is established by tissue biopsy, followed by immunohistochemistry and genetic studies (71). Further evaluation of the disease progression can be obtained by staging systems, such as international prognostic index (IPI) and combined Positron Emission Tomography – Computed Tomography (PET-CT) (73).

### Treatment Strategies for B-Cell Malignancies

For fit patients, the combination of chemotherapy with corticosteroids remains the first line treatment for most of the listed malignancies. The anti-CD20 monoclonal Ab rituximab will be added for patients with CLL, B-cell NHL, and ALL. Patients that are ineligible for chemotherapy will be treated with specific pathway-inhibitors, such as Bruton tyrosine kinase (ibrutinib), B-cell lymphoma 2 (bcl-2) inhibitors (venetoclax), proteasome inhibitors (bortezomib, carfilzomib) or immunomodulating agents (lenalidomide, pomalidomide). For MM and Hodgkin lymphoma, monoclonal Abs are currently approved in the relapsed setting: daratumumab is the monoclonal Ab that binds to CD38, while brentuximab-vedotin is an Ab-drug conjugate that recognizes CD30. Autologous stem cell transplantation (SCT) will be performed at diagnosis for patients with MM or at relapse for NHL patients.

The efficacy of the initial therapy is evaluated by specific disease parameters and by minimal residual disease (MRD) status. MRD evaluation being negative is a strong indicator of prognosis-free survival while being positive suggests potential relapse (74). In case of disease relapse, a second line therapy is applied. Depending on the cancer type and relapse time, salvage therapy includes more and more specific pathway inhibitors that will be used in combination or in monotherapy. MRD determination has clinical implications in the treatment for ALL, where only MRD positive patients will undergo allogeneic SCT.

Current developments in immunotherapy, such as T-cell engaging BsAbs and chimeric Ag receptor T-cells (CAR-T), show promising results in the first clinical studies to enhance traditional approaches (75). The Ags expressed during B-cell development are illustrated in Figure 3. The clinical development of blinatumumab will be discussed later. CD19-binding CAR-T cells were recently approved by the Food and Drug Administration (FDA) and the European Medicines Agency (EMA) for the treatment of relapsed ALL and aggressive NHL.

## Bispecific Antibodies in Clinical Development

A selection of BsAbs in clinical development is shown in Table 3.

### Clinical Development for ALL, CLL, and/or NHL (CD19 – CD3)

CD19 is expressed from the early development of B-cells up to their differentiation into plasma cells. Targeting CD19 results in B-cell aplasia, which is considered as manageable since patients can receive intravenous Igs until the recovery of the B-cell lineage. When compared with other B-cell Ags, its broad expression profile and low negative regulation rate (76) makes CD19 a suitable target for B-cell malignancies. It is expressed in 80% of ALL cases, 88% of B-cell NHL and all cases of CLL (77).

Three main (anti-CD19 × CD3) BsAbs have been developed for the treatment of B-cell ALL: Blinatumumab, AMG103 (BiTE), MGD011 (dual-affinity re-targeting Ab: DART) and AFM11 (Tandem diabody: TandAb).

#### BiTE Anti-CD19 – CD3 (Blinatumomab; AMG103)

Blinatumomab is a BiTE with excellent cell-binding capacities due to its small size allowing a better tumor penetration compared to Igs (78). In humans, it was initially explored in relapsed/refractory (r/r) NHL and afterwards in ALL (79). It was approved by the FDA in December 2014 and the EMA in December 2015 for the treatment of r/r Ph-negative ALL (23, 80–83). However, it is currently being tested in clinical trials for other hematologic malignancies, such as NHL and MM.

Given its short half-life, blinatumomab is continuously administrated via an intravenous infusion, at a constant rate (after an increase in the initial dose) and by repeated cycles of 4 weeks, that are interrupted with 2 weeks without treatment (23). The observed side effects are mostly mild to moderate and occur during the first cycle. The treatment generally starts under vigilant monitoring with a lower dose during the first 7 days. The most commonly observed adverse effects are chills, pyrexia, constitutional symptoms and reversible neurological events, such as tremors, seizures, aphasia, and ataxia. Furthermore, up to 70% of patients had symptoms of a transient CRS (84). In order to minimize these effects, premedication with dexamethasone is required on the first day of each cycle and on the first day of any dose increase (5, 23).

Blinatumomab is currently in Phase I and II clinical trials in combination with monoclonal Abs (mAbs) targeting inhibitory checkpoints, such as programmed cell death protein 1 (PD-1) and cytotoxic T lymphocyte antigen 4 (CTLA-4) (Table 4).

**TABLE 4:** Clinical trials of BsAbs in combination with different immunotherapeutic strategies (selected trials).

Names (Sponsors)	Targets	Diseases indication	Phase (NCT#)
**Combinations with immune modulators**			
Combination of Blinatumomab and Nivolumab (anti-PD-1 mAb) +/− Ipilimumab (anti-CTLA4 mAb) [National Cancer Institute (NCI)]	CD3 × CD19 × PD-1 (x CTLA4)	B-ALL	Phase I (NCT02879695)
Combination of Blinatumomab and Pembrolizumab (anti-PD-1 mAb) (Merck Sharp & Dohme Corp., Amgen)	CD3 × CD19 × PD-1	B-ALL	Phase I/II (NCT03160079)
Combination of Blinatumomab and Pembrolizumab (anti-PD-1 mAb) (Amgen)	CD3 × CD19 × PD-1	NHL	Phase I (NCT03340766)
Combination of Blinatumomab and (anti-PD-1 mAb) (City of Hope Medical Center)	CD3 × CD19 × PD-1	ALL	Phase I/II (NCT03512405)
Combination of Blinatumomab and Pembrolizumab (anti-PD-1 mAb) (Children’s Hospital Medical Center, Cincinnati)	CD3 × CD19 × PD-1	B-cell lymphoma and leukemia	Phase I (NCT03605589)
Combination of BTCT4465A and Atezolimumab (anti-PD-L1 mAb) (Genentech)	CD3 × CD20 × PD-L1	CLL, NHL	Phase I (NCT02500407)
Combination of REGN1979 and REGN2810 (cemiplimab: anti-PD-1 mAb) (Regeneron Pharmaceuticals)	CD3 × CD20 × PD-1	Lymphoma	Phase I (NCT02651662)
Combination of REGN1979 and REGN2810 (anti-PD-L1 mAb) (Hoffmann-La Roche)	CD3 × CD20 × PD-L1	NHL	Phase I (NCT03533283)
**Combination with mAb**			
Combination of JNJ-64407564/JNJ-64007957 and Daratumumab (Janssen)	CD3 × BCMA or GPRC5D × CD38	MM	Phase I (NCT04108195)
**Combination with ADC**			
Combination of BTCT4465A and Polatuzumab vedotin (anti-CD79b × MMAE) (Hoffmann-La Roche)	CD3 × CD20 × ADC	B-cell NHL	Phase I (NCT03671018)

*Data available as of November 05, 2019. Molecules are classified based on target antigens. ADC, antibody-drug conjugate; BCMA, B-cell maturation antigen; CTLA4, cytotoxic T-lymphocyte-associated protein 4; GPRC5D, G protein-coupled receptor family C group 5 member D; mAb, monoclonal antibody; MMAE, monomethyl auristatin E; PD-1, programmed cell death 1; PD-L1, programmed cell death 1 ligand.*

#### DART CD19 – CD3 (MGD011)

MGD011 (duvortuxizumab) is a CD19 × CD3 DART with a silenced, human IgG1 Fc domain. The presence of this Fc domain prolongs its circulating half-life (approximately 14.3 to 20.6 days), similar to conventional mAbs, allowing for an administration every 2 weeks (85). The humanized Ab arms have a 10-fold greater affinity for CD19 than for CD3, thereby enabling preferential binding to target cells, while minimizing the engagement of CD3 in the absence of target cells. Although the preclinical results in murine NHL models was promising, the clinical development of MGD011 was discontinued early due to high levels of neurotoxicity observed in a Phase I study on the treatment of B-cell malignancies (NHL, CLL, and NCT02743546) (85).

#### TandAb CD19 – CD3 (AFM11)

AFM11 is a tetravalent bispecific TandAb with two binding sites for CD3 and two for CD19. This structure increases the binding affinities for CD19 and CD3 by approximately 5- and 100-fold, respectively, compared to those of BiTE. Furthermore, AFM11 potency is not correlated with CD19 density on the surface of the target cell (86). This BsAb was tested in phase I studies for the treatment of ALL (NCT02848911) and r/r NHL (NCT02106091). These two clinical trials were suspended due to neurological side effects that caused the death of one patient and life-threatening toxicities in two others. Therefore, the risk/benefit profile was not favorable with the dosing regimens studied, putting an end to these two clinical studies.

### Clinical Development for ALL, CLL, and/or NHL (CD20 – CD3)

The CD20 Ag is expressed exclusively on mature B-cells and not on B-cell precursors, stem cells and plasma cells. It is also observed on the surface of malignant B-cells: more than 95% of B-cells in NHL and other B-cell malignancies express CD20.

#### TriFab CD20-CD3 (FBTA05)

FBTA05 (Lymphomun) has a TriFAb format; the third functional site is the Fc region which provides an additional capacity to recruit accessory cells bearing the Fcγ receptor (FcγR) (macrophages, dendritic cells, NK-cells and neutrophil granulocytes) (87). Promising responses have already been observed in pediatric patients (88, 89), but details on its further development or its current status are not clear.

#### IgG4-Based CD20 – CD3 (REGN1979)

REGN1979 is a fully humanized bispecific IgG4 Ab designed to resemble natural human Abs (90). As a result, this construct has the advantages of native Abs, such as stability, low aggregation propensity, low immunogenicity and good pharmacokinetics. This BsAb induces prolonged B-cell depletion in the peripheral blood as well as in lymphoid organs in preclinical models (90). In a phase I study on r/r NHL, administration of BsAb resulted in an overall response of 100% in follicular lymphoma and provided a complete response in two patients who did not respond to CAR T-cell therapy (91).

#### IgG1-Based CD20 – CD3 (Mosunetuzumab)

Mosunetuzumab (or BTCT4465A) is a another full-length, humanized IgG1 molecule with an almost native Ab structure using KiH technology. The first clinical results with mosunetuzumab were recently reported: In the patients with r/r aggressive NHL, the objective response rate (ORR) was 37.1%, with a complete response rate of 19.4%. Higher response were seen in the group with indolent NHLs with an ORR of 62.7% and complete response (CR) rate of 43.3% (92).

#### CD20-CD3 (RG6026)

RG6026 is a BsAb that binds to CD20 and CD3 in a 2:1 format, providing better affinity for the tumor Ag. The CD3 binding arm is fused directly to one of the CD20 binding arms via a short flexible linker. RG6026 also has a modified heterodimeric Fc region that prevents binding to FcγRs, while binding to the neonatal Fc Receptor is maintained, which results for an extended circulatory half-life (93). It showed significant *in vitro* and *in vivo* activity even on cells expressing low levels of CD20, it remains active in the presence of competing anti-CD20 antibodies and can potentially bypass the resistance to rituximab (94). Furthermore, its cytotoxicity activity has been observed even at very low effector:target ratios (95).

Clinical trials are underway to evaluate the efficacy of these different anti-CD20 × anti-CD3 BsAbs (Table 3). Several of these CD20-targeting BsAbs (Mosunetuzumab, REGN1979, and RG6026, etc.) are currently in Phase I clinical trials in combination with monoclonal Abs targeting the PD-1 inhibitory checkpoint or its ligand, PD-L1 (Table 4).

### Clinical Development for Lymphoma (CD30 – CD16A)

AFM13 is a tetravalent BsAb in the TandAb format without Fc domain (24). Therefore, it has two binding sites for CD30, located between two binding sites for CD16A. The center of the molecule interacts with the CD30 Ag, whereas the effector cell binds to both ends of the molecule. It is used to direct NK-cell toxicity to CD30-expressing lymphoma cells. It has been shown that AFM13 activates NK-cells only after binding to CD30 (94). AFM13 has shown signs of activity in a Phase I study, as well as effective NK-cell activation and a decrease in soluble CD30. Moreover, it has been well tolerated and may even be better tolerated than T-cell based BsAbs (24). AFM13 is currently in phase II clinical development (Table 3).

### Clinical Development for MM (CD38 – CD3)

The uniformly overexpressed CD38 Ag is the most widely studied target in the treatment of MM (96). Intriguingly, it is also expressed by many other hematopoietic cells, but treatment with the monoclonal Ab daratumumab is safe and without major side effects (96).

Several humanized anti-CD38/CD3 XmAb BsAbs and with different affinities for CD38 and CD3, were simultaneously evaluated during the preclinical stage. The best *in vitro* and *in vivo* results were obtained with AMG424. Although it has a lower affinity for CD3 to prevent an uncontrolled CRS in the presence of soluble CD38, it shows strong anti-tumor effects (97). Given that CD38 is also expressed by T-cells, a fratricide problem could interfere with the activity of AMG424. A Phase I Study (NCT03445663) evaluating the safety, tolerability, pharmacokinetics, pharmacodynamics, and efficacy of AMG 424 in recurrent/refractory Multiple Myeloma (r/r MM) began in 2018 and will end in 2022.

GBR 1342 is another anti-CD38/CD3 BsAb that is developed by Glenmark. It contains a complete Fc domain with a reduced effector function. In preclinical studies, GBR1342 showed a more potent anti-cancer effect than the anti-CD38 mAb daratumumab. It efficiently recruited T-cells and induced CD38 + cell depletion in the blood and especially the bone marrow (98). A Phase I study (NCT03309111) started in October 2017 evaluating the safety and tolerability of GBR 1342.

### Clinical Development for MM (BCMA – CD3)

BCMA is a membrane Ag expressed by malignant plasma cells as well as plasmacytoid dendritic cells. In contrast, it is not expressed on naive B-cells, CD34 + hematopoietic cells or any other normal tissue cells (99–101). BCMA has several advantages making it a highly studied target as part of the treatment for MM. First, BCMA is highly expressed by MM cells, as well as in patients with poor prognosis. Second, a rapid re-emergence of B-cell immunity after the end of the anti-BCMA treatment would be possible since this Ag is not expressed early in B-cell development. Third, the lack of BCMA expression in other bone marrow populations prevents off-tumor toxicities.

Several BsAbs are currently in clinical trials to evaluate their efficacy primarily in patients with advanced MM who have relapsed or are refractory to standard treatment (Table 3).

#### BCMA-CD3 BiTEs (AMG420 and 701)

AMG420 (or BI 836909) is a BiTE that has a short half-life time and therefore must be administered intravenously for 4 weeks followed by 2 weeks treatment-free. While AMG420 induces potent lysis of BCMA-positive MM cells *in vitro* and *in vivo*, BCMA-negative cells were not affected. Accordingly, clinical trials started for the treatment of r/r MM in 2015 (NCT02514239) and in 2019 (NCT03836053) (102). In a phase I study including 42 refractory MM patients, a high response rate of 70% was observed including 50% MRD-negative complete responses. The most common side effects were infections and polyneuropathy. AMG701 is a half-life–extended BiTE that contains the single-chain variable fragments of AMG420. It is suitable for once-weekly dosing and is currently tested in a phase I trial. Comparison of the observed responses and toxicities, allows to study the clinical implications of such a half-life extension.

#### BCMA-CD3 CrossMabs (EM801, CC-93269)

EM801 is a CrossMab in the 2 + 1 format. Its prolonged half-life due to maintenance of the Fc region allows for a convenient weekly intravenous treatment. Nonetheless, it is eliminated from the circulatory system within 1 to 2 months of treatment discontinuation. EM801 achieved lysis of 90% of myeloma cells after 48 h with a very low E:T ratio (103). The first results of a related molecule, EM901/CC-93269 (ENgMab/Celgene), on 30 r/r MM patients were recently presented: clinical activity was seen at higher doses of the drug with almost 90% of the patients responding at the highest dose. 76% of patients developed a CRS which was severe (> Grade 3) in one patient (104).

#### IgG2a-Based BCMA-CD3 (PF-06863135)

PF-06863135 (PF-3135) is a humanized BsAb using a IgG2a backbone with mutations in the Fc part that promote heavy chain heterodimer formation and reduce Fcγ receptor binding (105). This BsAb showed potent anti-myeloma activity in both *in vitro* and *in vivo* models and its toxicity profile in cynomolgus monkeys was acceptable (105). PF-06863135 is currently undergoing a Phase I study to assess its safety and tolerability (NCT03269136) (106).

### Clinical Development for MM (FcRL5 – CD3 and GPRC5D – CD3)

Two new targets have recently emerged as part of the MM-related targets: Fc Receptor-Like 5 (FcRL5) and G-protein coupled receptor family C group 5 member D (GPRC5D).

The first (also known as FcRH5, IRTA2, or CD307) is a specific and exclusive surface marker of the B-cell lineage. Its expression is detected starting from the pre-B-cell stage (107). However, unlike other B-cell-specific surface proteins, FcRL5 expression is preserved in normal and malignant B-cells (including plasma cells). This suggests a potential broader applicability of this target in B-cell malignancies, such as chronic lymphocytic leukemia, mantle cell lymphoma, diffuse large B-cell lymphoma, and follicular lymphoma (107, 108).

In contrast, GPRC5D is expressed on the surface of malignant cells involved in multiple myeloma without being expressed at appreciable levels by normal hematopoietic cells, such as T-cells, NK-cells, monocytes, granulocytes and bone marrow progenitors, including hematopoietic stem cells (109). High mRNA expression of GPRC5D was observed in patients with MM, whereas only low expression was detected in normal tissues. Its mRNA expression was also significantly correlated with poor overall survival rates (110). As a result, its very limited expression profile makes it a suitable target in MM treatment.

Two BsAbs have been developed against these two targets and are currently in a phase I clinical trial: RG6160 which targets FcRL5 (NCT03275103) and the DuoBody JNJ-64407564 which targets GPRC5D (NCT03399799) (Table 3). Both showed *in vitro* and *in vivo* B-cell depletion and tumor growth suppression in myeloma models (35, 109).

## Concerns in Clinical Development

### Cytokine Release Syndrome (CRS)

CRS is a potentially fatal systemic inflammatory reaction that is observed after the infusion of immunotherapeutic agents (monoclonal Abs, BsAbs, and CARs). Although our understanding of CRS is incomplete, different immune populations including T-lymphocytes, monocytes and macrophages are activated, all resulting in a mass production of inflammatory cytokines, particularly interleukin (IL)-6 and interferon (IFN)-γ (111). Although the immunological cascade is initiated by T-cell activation, this massive systemic production of toxic cytokines is mainly due to monocyte and macrophage activation. T-cell IFN-γ, macrophage IL-6, IL-10 and tumor necrosis factor alpha (TNF-α) seem to cooperate to facilitate this cytokine release (112). In addition, IL-6 has been shown to play a central role in humans and mice in the development of CRS (111, 113). Patients presenting CRS usually develop mild fatigue, fever, chills, headache, arthralgia, or even more serious life-threatening problems, such as hypotension, tachycardia, vascular leaks and circulatory collapse during or immediately following administration of the drug.

In general, signs and symptoms of CRS only appear during the first cycle of the drug, and not later during subsequent administrations. This CRS is not implicated in the mechanisms of action of T-cell directed immunotherapies (114), as the response to treatment is unaffected by the severity of CRS (115). A mitigation strategy based on corticosteroids and IL-6 blockade has been proposed to minimize the release of toxic cytokines (112).

An alternative way to avoid CRS-related problems is to dissociate tumor cell destruction and cytokine release. There are two distinct thresholds for T-cell activation based on the number of TCR- peptide-MHC (pMHC) complexes formed (116). The formation of two TCR-pMHC complexes is sufficient between a T-cell and an Ag-presenting cell, to trigger T-cell-mediated cell lysis. On the other hand, 10 TCR-pMHC complexes are required for the formation of a complete immune synapse and cytokine secretion. Thus, adjusting the binding characteristics for the CD3-binding arm, a BsAb could more closely mimic the natural TCR-pMHC induced T-cell activation (117). Consequently, new CD3-binding Abs have been generated that bind to multiple epitopes on CD3 with a wide range of affinities and agonist activities. Functional studies were realized with BsAbs that integrated the different CD3-binding domains. A BsAb with a new T-cell-engaging domain could be created that elicited strong *in vivo* tumor cell killing and low levels of cytokine release (118).

### Neurotoxicity

Neurotoxicity is the second most common adverse effect observed with different BsAbs. Symptoms may range from subtle changes in personality to tremors, vertigo, confusion, and focal neurological symptoms to more serious episodes of encephalopathy, ataxia, cerebellar alteration, convulsions and delirium (23). The pathophysiology of these neurotoxic effects still has not been determined but, as in CRS, inflammatory cytokines appear to be involved (119).

Grade 3 or higher neurotoxicity occurs in approximately 10 to 20% of the patients treated with blinatumomab (5, 120). However, in most cases, the neurological side effects were reversible after stopping the BsAb perfusion and initiation of corticosteroids. Furthermore, grade 3 or higher neurological events were avoided using a progressive dosing regimen and the prophylactic administration of dexamethasone. Although the application of steroids relieves the central nervous system symptoms, it could potentially hamper the immune response. While reduced levels of inflammatory cytokines were produced by dexamethasone-treated T-cells, there was no inhibitory effect of dexamethasone on the cytotoxic capacities of T-cells observed (121). This indicates that dexamethasone does not interfere with the therapeutic efficacy of BsAbs.

### Administration Route

The most commonly used administration route for BsAbs is intravenous (IV) perfusion. Although it has advantages in terms of pharmacokinetics and pharmacodynamics, it has certain drawbacks with regards to patient convenience, access to therapeutic targets and cost of treatment. The reduced half-life time of some BsAbs results in either more frequent administrations or continuous infusion (11, 12). On the other hand, the addition of an Fc domain facilitates the BsAb purification, improves solubility and stability, and molecule’s half-life (12). However, although BsAbs with an extended half-life may ease the logistics of administration, prolonged exposure could potentially increase the toxicity. Ongoing clinical trials will test this hypothesis and confirm or refute it.

### Resistance Mechanisms

#### T-Cell Exhaustion/Dysfunction

During cancer development, T-cells rapidly become dysfunctional due to persistent Ag-exposure. This reduces their proliferation capacity and their cytotoxic effector function. Moreover, several inhibitory receptors (such as PD-1, CTLA-4, T-cell immunoglobulin and mucin domain-3 (TIM-3), Lymphocyte-activation gene 3 (LAG-3), T-cell immunoglobulin and ITIM domain (TIGIT) are overexpressed by malignant cells (122, 123). Among them, the PD1/PD-L1 axis appears to be a central process in T-cell dysfunction (124). Targeting these inhibitory pathways is currently used to block immune suppressive signals coming from tumor cells and to prolong T-cell activation.

T-cell exhaustion is characterized by a progressive loss of function, such as proliferation, cytokine production, and cell lysis. T-cells do not become totally inactive, but fail to effectively eradicate cancer cells. Three distinct signals are normally required for optimal T-cell activation and proliferation. First, an Ag recognition via the TCRs is needed, followed by a costimulation and a cytokine release by the T-cells, which is required for their expansion. BsAb only provide the first signal. However, BiTEs and many other Ab formats may trigger the formation of an effective immunological synapse, abolishing the need for co-stimulation (125). Co-activation of T-cells through CD28 or 4-1BB, will increase the activation of T-cells by BsAbs (126, 127). Regarding the third requirement, new BsAb constructs have been developed to include cytokine IL-15 (128). Moreover, as mentioned previously, the blockade of PD-1 or its ligand, PD-L1, can successfully reactivate T-cell function.

Unfortunately, most patients do not maintain sustainable responses to this treatment. The lack of a sustainable response can be at least partly explained by the presence of other inhibitory pathways in T-cells. Thus, the identification of resistance and evasion mechanisms as well as the understanding of the processes that direct and maintain the various dysfunctional T-cell states are still a major concern for enabling effective BsAb activity targeting T-cells, while avoiding potentially life-threatening autoimmune side effects (129).

#### Antigen Escape

Tumor cells can also downregulate a targeted Ag and circumvent immune recognition during treatment. For example, loss of CD19 has been observed in patients with ALL, contributing to progression of the leukemia in 10 to 20% of cases. Altered membrane traffic and export (130) as well as, acquired mutations and alternative splicing explain this loss of expression at the cell-surface, while its intracellular abundance is preserved (131). Alternative splicing can, for example, result in the loss of CD19 extracellular domain (131). This leads to a conformational change in the extracellular domain of CD19, while the loss of a chaperone molecule (CD81) can lead to the intracellular accumulation of CD19 (130).

Consequently, a potential strategy to control Ag escape is to combine the targeting of several Ags in order to generate T lymphocytes that can recognize several Ags expressed on the tumor cells. For instance, a clinical study evaluating the efficacy of an anti-CD19/anti-CD22 BsAb is currently ongoing (NCT02370160) (132) (Table 3).

#### Immunosuppressive Microenvironment

Another major concern is the possible involvement of tumor microenvironment factors, such as immunosuppressive regulatory T lymphocytes (Tregs). Given that BsAbs trigger T-cell activation via binding to the CD3 complex, other T lymphocyte cell subtypes, besides effector T lymphocytes, will also be activated (133). A high percentage of Tregs present in the tumor environment predicts a resistance to treatment. For example, Tregs, activated by blinatumomab, are able to suppress the proliferation of effector T-cells and the subsequent cell lysis. As a result, T-cell depletion prior to administration of blinatumomab may increase effectiveness for non-responding patients treated with blinatumomab (19).

#### Immune Checkpoint Receptor PD-1

PD-1 is a co-inhibitory receptor that acts as an immune checkpoint. It is used to attenuate immune responses by limiting the duration and intensity of the immune reaction. Tumor cells often express its ligand, PD-L1, to evade immune system attacks (134). It is an adaptive mechanism of immune escape in response to pro-inflammatory cytokines (135). A wide range of anti-PD-1 antibodies (nivolumab, pembrolizumab) or anti-PD-L1 antibodies (atezolizumab, durvalumab, avelumab) have been tested in mono- or in combination therapy (136). However, PD-L1 is widely expressed on healthy tissues and therefore, the efficacy of these blocking Abs can be reduced due to binding to PD-L1 positive normal cells. This may lead to blind activation of T-cells, including those involved in (auto)immune-related adverse events such as endocrinopathy (for example, thyroiditis), dermatitis, pneumonia, hepatitis, and colitis (137–139).

Immune modulation through PD-1 is one of the mechanisms of resistance to blinatumomab (140). While refractory leukemic blasts overexpressed PD-L1, T-cell exhaustion was observed with overexpression of PD-1. Combination of blinatumomab and the anti-PD-1 antibody Pembrolizumab enhanced T-cell function and induced an anti-leukemic response in a 12-year-old patient with refractory ALL (140). The activity of blinatumomab could also be restored by adding an anti-PD-L1 × CD28 BsAb that abolished the PD-L1 mediated resistance and even reverted the negative PD-L1 signaling into positive costimulation through CD28 on T-cells (141). The combined action of PD-1/PD-L1 blocking Abs and BsAbs inspired the design and initiation of clinical studies combining blinatumomab with checkpoint inhibition as summarized in Table 4. In order to improve the clinical benefit, BsAbs that simultaneously target two immune checkpoints have been developed. For example, the dual blockade of PD-1 and LAG-3 with monoclonal Abs further suppresses T-cell activation. For instance, an anti-PD-1/anti-LAG-3 DART, called MGD013, binds specifically to both PD-1 and LAG-3 (142). Blocking both pathways enhanced T-cell responses compared to those observed upon independent blockade of either the PD-1 or LAG-3 pathways alone. The BsAb KN046 is another that binds to PD-L1 on the tumor cells and to CTLA-4 expressed by the T-cells. However, the increase in anti-tumor activity has been associated with a significant increase in the number of adverse events due to over-activation of the immune system. Consequently, a new approach is currently being investigated. It consists in the deletion of the PD-1 pathway via high-affinity PD-1 binding, while inhibiting CTLA-4 with a low affinity binding arm. This construct inhibits CTLA-4 in double-positive T-cells while reducing the binding to peripheral T lymphocytes expressing CTLA-4, resulting in better tolerability (143).

#### The Co-stimulatory Receptor 4-1BB

4-1BB (CD137) is a potent co-stimulatory receptor that is upregulated on effector T lymphocytes including tumor infiltrating T-cells. Its stimulation improves cytotoxic function, as well as the induction of an immunological memory (144). In addition to its function on T-cells, it has been shown to improve the cytotoxic function of NK-cells (145). 4-1BB-binding monoclonal Abs are classified according to their agonistic capacities and Fc receptor affinities. While urelumab is a strong agonist and inducing signal activation without Fc receptor binding, the basal agonistic activity of utomilumab is weak but increases after Fc receptor crosslinking (146). The clinical development of these first-generation Abs was stopped: utomilulab showed only a reduced efficacy (although no major toxicities were seen) and urelumab showed efficacy but also severe liver toxicity (147, 148). Interestingly, new 4-1BB binding Abs have recently been created by adapting the level of intrinsic agonistic activity, the FcγR interactions, the IgG subclass and Ab affinities (146, 149). Another strategy to overcome the limitations of the first- generation Abs is the integration of 4-1BB-binding domains in BsAbs.

A few BsAbs containing a tumor Ag-binding fragment and a 4-1BB agonist have been developed (150–152). The main characteristic of these compounds is the lack of significant 4-1BB activation in the absence of tumor Ag binding, ensuring tumor-localized immune activation. For example, a BsAb that simultaneously targets 4-1BB and the CD19 tumor Ag was developed for systemic administration (153). Since additional mutations in the Fc region prevents Fcγ receptor cross-linking, the 4-1BB in this construct is only activated when cross-linked to CD19 and thus, hepatic toxicity is avoided (9). Another example of BsAb targeting checkpoint agonists is INBRX-105 (Inhibrx) which is directed toward PD-L1 and 4-1BB. While simultaneously suppressing inhibition via the PD-1 – PD-L1 axis, it is designed to only activate T-cells via 4-1BB in the tumor environment when it encounters PD-L1 (17).

#### Immune Checkpoint Receptor CD47

CD47 [Integrin-associated protein (IAP)] is ubiquitously expressed in normal tissues and can be found on mesenchymal stromal cells and blood cells, particularly erythrocytes and platelets, and is generally upregulated in cancers. When it binds to its ligand, the signal regulatory protein α (SIRPα) which is an inhibitory receptor on macrophages and dendritic cells, CD47 sends “don’t eat me” signals by inhibiting phagocytosis of tumor cells and triggering an immune evasion (154).

Hematological cancer cells overexpress CD47 in order to evade removal by phagocytes (macrophages and dendritic cells) (154, 155). As a consequence, both the innate and adaptive anti-cancer immune responses are suppressed. Therefore, CD47 neutralizing antibodies could improve tumor lysis by effector cells. However, CD47 is also widely expressed on normal cells (156). Thus, a general blockade of the CD47/SIRPα interaction may result in the removal of normal healthy cells and may be associated with toxicity.

Furthermore, the abundant expression of CD47 throughout the entire human body could eventually lead to the formation of “Ag sinks” that would prevent anti-CD47 antibodies from reaching the targeted tumor. To circumvent this problem, BsAbs with a low affinity for CD47 and a high affinity for a tumor Ag have been developed, which guarantee CD47 to be bound by BsAb only on tumor cells co-expressing both Ags. For example, a CD47 × CD19 BsAb (TG-1801, NI-1701, NovImmune, TG Therapeutics) induced increased phagocytosis by Fc and retained its activity in the presence of high amounts of non-tumor-associated CD47 (157). However, the functional Fc domains present in this BsAb can cause the off-target premature activation of Fc receptor (FcR)-expressing phagocytes, thereby causing systemic toxicity. Another BsAb format called RTX-CD47, targeting CD47 and CD20 without an Fc domain, triggered a significant phagocytic removal of both CD20 and CD47 malignant B-cells, but not cells expressing CD47 alone, while preventing toxicity associated with the presence of an Fc domain (158).

## Conclusion

As seen in different clinical trials, BsAbs are promising tools for the treatment of hematologic B-cell malignancies. They enable different mechanisms of action, each having its own advantages and disadvantages. Although anti-tumor effects are observed, their clinical translation is hampered by limiting side-effects, such as off-target effects, a reduced E:T ratio in pretreated patients, and pharmacological limitations. Therefore, combined expertise in immunology, pharmacology and Ab engineering is required to improve their efficacy. A number of approaches are currently being studied and include combinations with checkpoint inhibitors, chemotherapy and other existing treatments. The different platforms on which BsAbs are produced will further improve their anti-tumor activity. Looking at the variety of targets, indications, mechanisms of action and implicated companies, it is clear that BsAbs will become key players in the field of immunotherapy.

## Author Contributions

JC and ML contributed to the conception and design of this review. ML and MK wrote the first draft of the manuscript. All authors contributed to manuscript revision, read, and approved the submitted version.

## Conflict of Interest

The authors declare that the research was conducted in the absence of any commercial or financial relationships that could be construed as a potential conflict of interest.
